# Suitability of Thrombolysis for Patients With Acute Ischemic Stroke Complicated With Trousseau Syndrome

**DOI:** 10.3389/fnins.2020.00481

**Published:** 2020-06-12

**Authors:** Yujie Chen, Chundi Zhang, Xin Wang, Long Han, Shiguang Zhu, Yan Liu, Rui Wang, Ziyang Geng, Chenchen Ma, Ruiguo Dong

**Affiliations:** ^1^Department of Neurology, The Affiliated Hospital of Xuzhou Medical University, Xuzhou, China; ^2^Department of Orthopedics, The Affiliated Changzhou No.2 People’s Hospital of Nanjing Medical University, Changzhou, China

**Keywords:** Trousseau syndrome, acute ischemic stroke, rtPA intravenous thrombolysis, cerebral hemorrhage after thrombolysis, recurrence

## Abstract

Intravenous thrombolysis (IVT) improves functional outcome after acute ischemic stroke (AIS) and is the standard first-line treatment; however, it is associated with many complications, including cerebral hemorrhage. Cancer patients are susceptible to thrombotic events – collectively referred to as Trousseau syndrome (TS) – owing to their hypercoagulable state. Here, we describe the case of a 55-year-old male with a history of hypertension for over 10 years who underwent surgery for removal of a cancer of lower esophagus, with no subsequent treatment. Three months later, he was admitted to the emergency department of our hospital with sudden dizziness and incoherent speech. Brain computed tomography revealed multiple cerebral infarctions. The patient was treated by IVT with tissue plasminogen activator (rtPA) after the onset of symptoms, which improved by the end of the treatment. However, a few months later, he experienced a recurrence of cerebral infarction and hemorrhage, which has rarely been reported. The clinical course of this case suggests that the suitability of thrombolysis with rtPA in the acute phase of cerebral infarction complicated with TS should be carefully considered.

## Introduction

Ischemic cerebral infarction is a major cause of disability and mortality in adults worldwide (Shigematsu et al., 2015; Jönsson et al., 2018). Although patients are carefully screened to minimize bleeding risk before administration of intravenous thrombolysis (IVT) according to the contraindications set forth in clinical guidelines, post-thrombolytic intracerebral hemorrhage is almost inevitable and potentially fatal in 7–8% of patients (Szekely et al., 2018). One multicenter study reported that 3.4% of patients had remote parenchymal hemorrhage after IV injection of recombinant tissue plasminogen activator (rtPA) (Prats-Sanchez et al., 2016).

The French internist Armand Trousseau (1801–1867) was the first to propose a non-random association between hidden visceral cancers and a hypercoagulable state resulting in an increased risk of venous thrombosis (Trousseau, 1968). If a cancer patient suddenly experiences acute ischemic stroke (AIS), a diagnosis of Trousseau syndrome (TS) must be considered. In the current American Heart Association/American Stroke Association guidelines, there is no mention of IV rtPA treatment of AIS with TS (Powers et al., 2019), and there are few reports of cases of recurrent ischemic bleeding following IV rtPA injection in patients with TS.

## Case Description

The patient, a 55-year-old male with a 10-year history of hypertension, was admitted to the thoracic surgery department of our hospital for dysphagia. Chest enhanced computed tomography (CT) revealed cancer in the lower esophagus and cardia. 1 week later, cardia cancer surgery was performed, and pathological examination showed highly differentiated adenocarcinoma; pathological classification was T4N1M0. The patient recovered after the operation and was discharged from the hospital without additional radiotherapy and chemotherapy.

**FIGURE 1:**
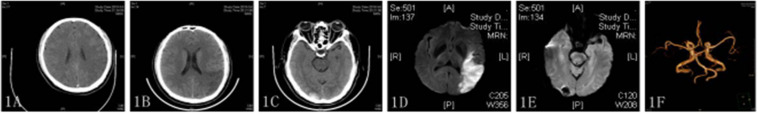
**(A–C)** CT image showing an indistinct gray matter boundary of the left frontal gyrus **(A)**; a large, low-density shadow in the left parietal occipital lobe **(B)**; and multiple high-density shadows on both sides of the occipital lobe **(C)**. **(D,E)** Diffusion-weighted imaging (DWI) showing a high-intensity signal in the left frontoparietal–temporal–occipital artery island **(D)** and low-intensity signal in the left cerebellar hemisphere **(E)**. **(F)** CT angiography showed no stenosis or occlusion.

Three months later on March 5, 2019 (2019/03/05), he experienced a sudden episode of dizziness, incoherent speech, and weakness of his right limb during rest. His vital signs were stable after admission, and electrocardiography (ECG) showed no atrial fibrillation. His National Institutes of Health Stroke Scale (NIHSS) score was 6. The results of first and subsequent coagulation function tests are shown in Table 1, and accompanying inflammatory indicators (CRP) are shown in Table 2. Head CT showed an ambiguous boundary between gray and white matter in the left parietal lobe (Figure 1A), and chest CT showed changes in the area of gastric tumor resection – namely, multiple enlarged lymph nodes near the descending aorta. Other blood biochemical indices were normal. The patient was treated by IVT with 0.6 mg/kg rtPA 156 min after symptom onset. By the end of thrombolysis, the right limb weakness had improved, with an NIHSS score of 4; 24 h after thrombolysis (2019/03/06), CT showed multiple infarction in the left parietal lobe and multiple blood foci in the right occipital lobe (Figures 1B,C). At this time, the patient did not have obvious discomfort, and the dizziness and speech symptoms were slightly improved. Magnetic resonance imaging (MRI) performed on the 3rd day after thrombolysis (2019/03/07) revealed a large infarction area in the left temporoparietal lobe and hemorrhage in bilateral occipital lobes and the left cerebellar hemisphere (Figures 1D,E). A second coagulation function test was performed. Enhanced MRI showed that there was no tumor metastasis in the brain, and no atrial fibrillation or other arrhythmias were observed upon ECG examination. No thrombus was found by CT angiography (Figure 1F), and no obvious plaques were detected in the neck and lower extremities by color Doppler ultrasound. The patient has both cerebral infarction and cerebral hemorrhage, considering that antiplatelet drugs can aggravate the risk of bleeding, so he did not have antiplatelet treatment during the first hospitalization. The patient’s blood was hypercoagulable, suggesting cerebral embolism; however, a series of tests did not reveal the location of the thrombus. Therefore, a diagnosis of TS with advanced gastric cancer was made. The symptoms of dizziness and speech incoherence improved (NIHSS score of 4), and the patient was discharged from the hospital.

**TABLE 1:** Coagulation parameters for the patient described in this study.

Coagulation parameter	Range of normal values	First 2019-03-05	Second 2019-03-07	Third 2019-04-10	Fourth 2019-04-18	Preoperative 2018-12-04
FIB (g/L)	2.00–4.00	1.54	1.15	0.96	11.3	2.55
D-Dimer (μg/ml)	0.00∼0.50	>10.00	7.53	6.29	9.99	0.2
FDP (mg/L)	<5.00	33.60	34.2	21.8	31.9	2.1
PT (s)	9.0–13.0	11.9	11.8	12	11.3	10.1
aPTT (s)	25.0–31.3	22.1	25.5	25.2	24.2	25.6
INR	0.9–1.2	1.03	1.03	1.04	0.98	0.88

*aPTT, activated partial thromboplastin time; FDP, fibrin degradation products; FIB, fibrinogen; INR, international normalized ratio; PT, prothrombin time.*

**TABLE 2:** CRP for the patient described in this study.

	Range of normal values	First 2019-03-05	Second 2019-03-07	Third 2019-03-21	Fourth 2019-04-10	Preoperative 2018-12-04
CRP (mg/L)	0.0–5.0	3.1	17.1	2.0	6.2	2.3

*CRP, C-reactive protein.*

**FIGURE 2:**
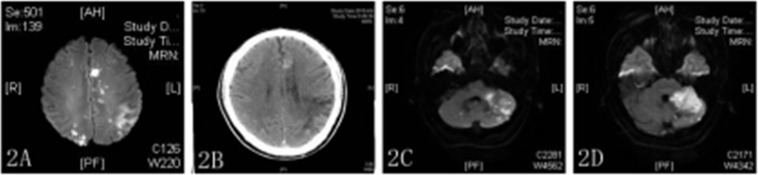
**(A,C,D)** DWI showed multiple high-density areas in the brain **(A)** and high-density shadows in bilateral cerebellar hemispheres **(C,D)**. **(B)** CT image showing a new high-density shadow.

One week after discharge (2019/03/21), the patient was re-admitted to the hospital with sudden right limb weakness and speech confusion. MRI showed a large new area of scattered infarctions (Figure 2A), and then he was given aspirin 100 mg, clopidogrel 75 mg, and atorvastatin 40 mg. After treatment on day 13 after admission (2019/04/02), the patient showed improvement despite an episode of sudden dizziness and vomiting on day 4 (2019/04/06), and was transferred to the rehabilitation department for further treatment. The transformation of bleeding after a new infarction was observed by head CT (Figure 2B). ECG revealed no obvious abnormalities, and the patient was transferred to our department for further treatment on 2019/04/08. On day 3 after admission (2019/04/10), MRI revealed new ischemic foci in the left cerebellum and absorption of hemorrhage in the right occipital lobe (Figures 2C,D). A third coagulation function test was performed. The patient had a history of recurrent cerebral infarction and hemorrhage after infarction that was complicated with abnormal blood coagulation function. In consultation with the hematology department, the patient was treated with cryoprecipitate (6 U) and reexamined 2 days later (the fourth coagulation function test). Blood coagulation function was improved, and 6000 U low-molecular-weight heparin was administered to the patient by subcutaneous injection along with oral administration of Rivaroxaban (15 mg) daily for 3 days. On 2019/04/24, the patient was in a stable condition and as the brain CT showed that the bleeding focus had been resorbed, he was discharged from the hospital.

On 2019/05/08, the patient had sudden obliquity of the mouth and incontinence and died 2 days later, 66 days after the first ischemic stroke. No autopsy was performed.

## Discussion

Since Armand Trousseau’s proposal 150 years ago that thrombotic events may be the first sign of concealed visceral malignancies (Zalatnai et al., 2018), the relationship between cancer and vascular infarction events has been investigated. Although the cause of TS remains unclear, recent studies have shown that the role of the tumor itself, the effect of hypercoagulability, and the side effects of tumor-related treatment may be the causes of the disease.

Trousseau syndrome is associated with elevated plasma D-dimer levels (Ferroni et al., 2012). Receiver operating characteristic curve analysis showed a D-dimer cutoff value of 2.0 μg/ml (odds ratio = 10.4; 95% confidence interval: 3.62–34.41; *P* < 0.0001), with a sensitivity of 71.1% and a specificity of 82.9% for distinguishing TS (Ito et al., 2018). D-Dimer level is higher in patients with cancer recurrence but decreases with tumor suppression, suggesting that the tumor causes blood hypercoagulation (Kuwahata et al., 2017). Our patient had a high D-dimer level, which confirmed the diagnosis of TS. Although no examination was performed to detect the occurrence of metastasis, chest CT revealed several enlarged lymph nodes near the descending aorta and metastasis to other tissues was suspected.

For the treatment of AIS in TS patients, there is no uniform standard. Cancer itself is related to hypercoagulability, which can lead to embolism. Thus, controlling the progression of cancer will play an important role in the development of TS. In such populations, treatment for anticoagulation or antiplatelet is still under discussion. On anticoagulant therapy, it is currently believed that heparin can inhibit coagulation activation and is the preferred drug for patients with TS (Wahrenbrock et al., 2003). Two articles published in New England in 2019 confirmed the role of oral anticoagulants rivaroxaban and apixaban in the treatment of cancer-related venous embolism (Carrier et al., 2019; Khorana et al., 2019). However, the anticoagulant value of these drugs currently applies only to TS patients with venous embolism. Navi et al. (2018) tried to compare the therapeutic effects of aspirin and heparin on cerebral infarction patients with TS, and they finally screened 20 patients. After follow-up, no significant difference was found in the cumulative incidence of major bleeding, thromboembolic events, and survival rates between the two groups. A recent study comparing the efficacy of rivaroxaban and aspirin in secondary prevention of AIS in TS patients (Martinez-Majander et al., 2020) found that patients with a history of cancer and no history of cancer had similar rates of recurrent ischemic stroke and all-cause mortality during aspirin and rivaroxaban treatment, but aspirin was safer for major bleeding. From the above experiments, it is still difficult to conclude whether TS patients should choose anticoagulant or antiplatelet therapy. After infusion of cryoprecipitate to correct abnormal blood coagulation function, our patient was treated with low-molecular-weight heparin as anticoagulant along with dabigatran. This treatment has not been documented in previous literature and is a personalized remedy based on the patient’s condition.

Alteplase is the first rtPA or tPA approved by the United States Food and Drug Administration for the treatment of thromboembolic diseases (Peters and Paciullo, 2015); it is currently the most commonly used agent for these conditions as its high fibrin specificity mitigates non-specific systemic effects and hemorrhagic complications. Clinical and experimental evidence has shown that rtPA exacerbates ischemic endothelial injury and blood–brain barrier disruption (Suzuki et al., 2016) and thus increases the risk of bleeding. In recent years, several large-scale clinical trials have evaluated the effect of rtPA on thrombolysis in TS patients (Murthy et al., 2013; Owusu-Guha et al., 2019; Weeda and Bohm, 2019). Concluding that the mortality of patients with TS after rtPA thrombolysis has not significantly increased, only the risk of bleeding in individual patients was higher than that in patients without a history of cancer. From these clinical trials, TS should not be a contraindication for rtPA thrombolysis. Our patient has repeated cerebral hemorrhage and cerebral infarction after rtPA thrombolysis, which is relatively rare in previous studies.

With the continuous improvements in medical care, cancer patients are living longer and the probability of acute cerebral infarction events in these patients is increasing. There is an ongoing debate among clinicians on the management of this patient population – i.e., whether thrombolysis, thrombectomy, anticoagulation, or antiplatelet therapy is most suitable.

## Conclusion

Although our patient met the standard of diagnosis for thrombolysis, he experienced repeated cerebral infarctions and bleeding after treatment. What made him go through this, we do not know, and we are guessing it is related to a change on coagulation function, which, of course, needs to be further tested.

## Data Availability Statement

All data containing relevant information to support the study findings are included in the article.

## Ethics Statement

All procedures were reviewed and approved by The Xuzhou Medical College Affiliated Hospital Office of Clinical Investigation and institutional review board. Written informed consent was obtained from a relative of the patient for publication of this article and any accompanying images.

## Author Contributions

YC, RD, CZ, and LH conceived and designed the study. YC, SZ, and YL acquired the data. YC, RW, and ZG analyzed and interpreted the data. All authors contributed to the writing of the manuscript, and read and approved the final version.

## Conflict of Interest

The authors declare that the research was conducted in the absence of any commercial or financial relationships that could be construed as a potential conflict of interest.
